# Causal relationship and shared genes between air pollutants and amyotrophic lateral sclerosis: A large‐scale genetic analysis

**DOI:** 10.1111/cns.14812

**Published:** 2024-07-05

**Authors:** Zhihao Li, Jie Wen, Wantao Wu, Ziyu Dai, Xisong Liang, Nan Zhang, Quan Cheng, Hao Zhang

**Affiliations:** ^1^ Department of Neurosurgery The Second Affiliated Hospital, Chongqing Medical University Chongqing China; ^2^ Department of Neurosurgery, Xiangya Hospital Central South University Changsha China; ^3^ National Clinical Research Center for Geriatric Disorders, Xiangya Hospital Central South University Changsha China; ^4^ Department of Oncology, Xiangya Hospital Central South University Changsha China; ^5^ College of Life Science and Technology, Huazhong University of Science and Technology Wuhan China

**Keywords:** air pollution, amyotrophic lateral sclerosis, C9orf72, Mendelian randomization, TMEM175, TWAS, USP35

## Abstract

**Objective:**

Air pollutants have been reported to have a potential relationship with amyotrophic lateral sclerosis (ALS). The causality and underlying mechanism remained unknown despite several existing observational studies. We aimed to investigate the potential causality between air pollutants (PM2.5, NO_X_, and NO_2_) and the risk of ALS and elucidate the underlying mechanisms associated with this relationship.

**Methods:**

The data utilized in our study were obtained from publicly available genome‐wide association study data sets, in which single nucleotide polymorphisms (SNPs) were employed as the instrumental variantswith three principles. Two‐sample Mendelian randomization and transcriptome‐wide association (TWAS) analyses were conducted to evaluate the effects of air pollutants on ALS and identify genes associated with both pollutants and ALS, followed by regulatory network prediction.

**Results:**

We observed that exposure to a high level of PM2.5 (OR: 2.40 [95% CI: 1.26–4.57], *p* = 7.46E‐3) and NOx (OR: 2.35 [95% CI: 1.32–4.17], *p* = 3.65E‐3) genetically increased the incidence of ALS in MR analysis, while the effects of NO_2_ showed a similar trend but without sufficient significance. In the TWAS analysis, TMEM175 and USP35 turned out to be the genes shared between PM2.5 and ALS in the same direction.

**Conclusion:**

Higher exposure to PM2.5 and NO_X_ might causally increase the risk of ALS. Avoiding exposure to air pollutants and air cleaning might be necessary for ALS prevention.

## Graphical abstract

Causality between PM2.5 and amyotrophic lateral sclerosis has been identified via two‐sample Mendelian randomization for the first time, with single nucleotide polymorphisms from genome‐wide association study serving as instrumental variables. C9orf72 turned out to be the top gene in the transcriptome‐wide association analysis. The figure was created by Biorender.com.

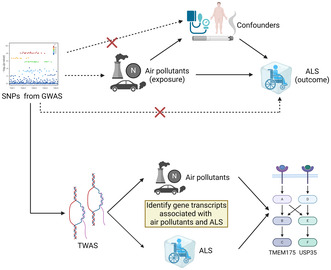

## 1 INTRODUCTION

Amyotrophic lateral sclerosis (ALS) is a rare but fatal neurodegenerative disease with an annual incidence of 1–2.6/100,000 persons.^1^, ^2^ The lifetime risk of ALS is estimated to be 1 in 400, and less than 10% of patients survive beyond 10 years.^3^, ^4^ ALS manifests as upper motor neuron and lower motor neuron dysfunction, resulting in progressive muscle weakness, atrophy, spasticity, paralysis, and respiratory failure.^5^, ^6^, ^7^ In ALS, available treatments only prolong life expectancy and maximize the quality of life. Therefore, there is an urgent need to prevent and manage this devastating disease. ALS can be classified into two main categories: sporadic ALS (sALS) and familial ALS (fALS). The majority of ALS cases are sporadic, meaning they occur without a clear family history, while about 10% of cases are fALS.^8^ Although studies of genetic variation in fALS help us make significant strides in uncovering the underlying mechanism,^8^, ^9^ most cases of ALS are sporadic with no clear factors, which may be triggered by the combination of genetic predisposition, environmental exposure, and the passage of time. This widespread agreement is known as the gene‐time‐environment hypothesis.

Research into the environmental exposome has shed light on factors potentially associated with amyotrophic lateral sclerosis (ALS). A meta‐analysis has summarized ALS's environmental risk factors, including exposure to heavy metals, organic chemicals, electric shocks, and physical injuries.^10^ While this analysis did not explore air pollution's impact on ALS, the significance of air pollutants is increasingly recognized in various diseases, including respiratory^11^ and cardiovascular diseases,^12^ as well as neurological disorders.^13^, ^14^ Therefore, air pollution's connection to ALS merits closer investigation in the context of these findings.

The causality between atmospheric pollution and ALS remained unknown despite multiple observational studies. For example, a study by Meinie et al. from the Netherlands involving 917 ALS patients and 2662 controls found a positive association between prolonged exposure to air pollutants from traffic sources and a higher chance of developing ALS.^15^ A recent Bayesian hierarchical analysis study similarly confirmed a highly positive correlation between ALS and elemental carbon concentration.^16^ But these studies above were limited by the inherent defects of observational studies, such as confounders and reverse causation, making it difficult to establish the causality. There is a pressing need to establish a causal relationship between air pollution and ALS.

We propose a two‐sample Mendelian randomization (TSMR) to address this issue to investigate the potential causality between air pollution and ALS. The fundamental principle of Mendelian randomization (MR) relies on the instrumental variants (IVs) analysis to make causal estimates. Three assumptions are required for MR: (i) IVs are strongly associated with the exposure; (ii) IVs are not associated with confounders from exposure to outcome and (iii) IVs act on the outcome only via the exposure. It is usually implemented using single nucleotide polymorphisms (SNPs) as IVs, which follow Mendel's laws of random assortment of genotypes in the natural world to mimic the design of a randomized controlled trial. Treating genetic variants as instrumental variables, which are presumed to be allocated randomly before birth, minimizes the potential influence of environmental factors. Moreover, as these genetic variants are established well before the onset of the disease, issues pertaining to residual confounding and reverse causation, commonly encountered in conventional observational studies, are effectively addressed.^17^, ^18^

With genome‐wide association studies (GWAS) providing existing summary statistics, MR has been extensively applied across diverse research domains. Several earlier studies employed MR to look into the connection between air pollution and different health outcomes.^19^, ^20^, ^21^, ^22^, ^23^, ^24^, ^25^, ^26^, ^27^, ^28^, ^29^, ^30^, ^31^ Yi et al., for example, conducted a TSMR analysis reporting a causal link between air pollution and neurodegenerative disorders (Alzheimer's disease and Parkinson's diseases).^24^ Wang et al. demonstrated the causal evidence that air pollution might cause multiple cancer types by MR.^20^ However, the causal relationship and underlying biological mechanisms between air pollution and ALS remains largely unexplored. Herein, we performed TSMR with existing GWAS data to assess our hypotheses that air pollution exposure may be causally linked to ALS. Furthermore, we conducted TWAS analysis based on the results of our MR, managing to explore possible mechanisms.

## 2 METHODS

**FIGURE 1:**
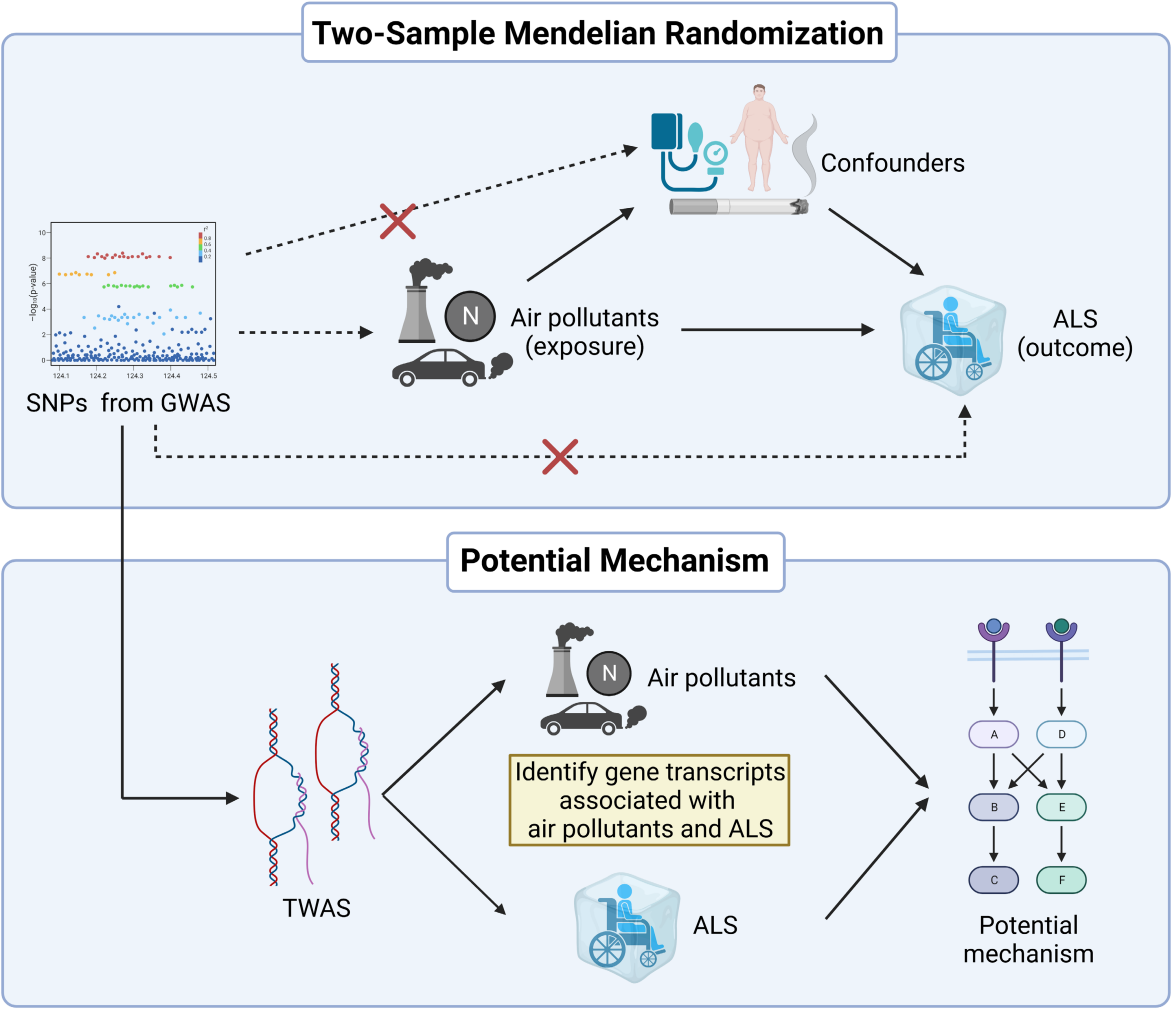
. Flowchart and study design of TSMR and TWAS. SNPs from publicly available GWAS data sets were selected as IVs based on their strong correlation with the exposure and independence from confounding factors. These IVs were required to influence the outcome solely through exposure, ensuring the credibility of the MR analysis. Furthermore, the GWAS data were converted into TWAS format to identify gene transcripts associated with air pollutants and ALS. The figure was created by Biorender.com. GWAS, genome‐wide association study; IVs, instrumental variants; SNP, single nucleotide polymorphism; TSMR, two‐sample Mendelian randomization; TWAS, transcriptome‐wide association.

The flowchart of the study is shown in Figure 1.

### 2.1 Data sources

The data utilized in our study were obtained from publicly available genome‐wide association study (GWAS) data sets and, therefore, do not require ethical approval or informed consent. All included GWAS data sets consisted of participants of European ancestry (Table S1), with no restrictions on gender, income, or education.

GWAS of exposure to air pollutants (PM2.5, NO_X_, NO_2_) were obtained from the UK Biobank (www.ukbiobank.ac.uk).^32^ The level of air pollutants in the UK was estimated using a land‐use regression model for the annual average 2010. The mean PM2.5 concentration was 9.99 ± 1.06 μg/m^3^, ranging from 8.17 to 21.31 μg/m^3^. The GWAS included 423,796 individuals and 9,851,867 SNPs. The mean NO_2_ concentration was 26.71 ± 7.58 μg/m^3^, ranging from 12.93 to 108.49 μg/m^3^, and the mean NO_X_ concentration was 44.11 ± 15.53 μg/m^3^, ranging from 19.74 to 265.94 μg/m^3^. The GWAS for NO_2_ and NO_x_ included 456,380 individuals and 9,851,867 SNPs.

The GWAS for ALS were obtained from the latest and largest meta‐analysis by van Rheenen et al.,^33^ which included 27,250 cases with familial or sporadic ALS and 110,881 control subjects. The participants of these GWAS were all European descent from European countries and the United States. The ALS cases in this large‐scale meta‐analysis were derived from independent cohorts and diagnosed by the EI Escorial criteria.

### 2.2 Selection of instrumental variants

Three principles were followed to select IVs in this study.^34^ First, IVs were required to exhibit strong and independent correlations with the corresponding exposure. As there were few SNPs under the threshold of 5e‐8, we set a stringent threshold of *p* < 1e‐6 to identify SNPs that demonstrated a strong correlation with the exposures of interest as in previous studies.^17^ Next, we employed the PLINK algorithm, with LD <0.001 and <10 MB distance from the index variant, to perform clumping and select independent IVs. Additionally, SNPs with *F* statistics <10 were excluded to guarantee the robustness of the IVs. Second, IVs were required to be unrelated to potential confounding factors such as body mass index (BMI), blood pressure, and smoking behavior. We conducted SNP lookups in the PhenoScanner database (http://phenoscanner.medschl.cam.ac.uk) to exclude any SNP with known associations with these confounding factors. Last, IVs were expected to be independent of the outcome and exert their influence solely through exposure. Thus, SNPs with a significant correlation with the outcome were excluded.

### 2.3 Statistical analyses

#### 2.3.1 Two‐sample Mendelian randomization (TSMR)

For the TSMR, random effects inverse variance weighting (IVW) was used as the primary method.^76^ IVW entailed a weighted regression of IV effects on the outcome, assuming a constrained intercept of zero, thus offering optimal statistical power. However, in the presence of horizontal pleiotropy, the outcome could be influenced by causal pathways other than the exposure itself.^35^ Hence, we employed the additional methods (weighted median, MR‐Egger, and Mendelian Randomization Pleiotropy RESidual Sum and Outlier [MR‐PRESSO]), which demonstrated relative robustness against horizontal pleiotropy, although with a partial sacrifice of statistical power.^36^, ^77^, ^78^, ^79^ The weighted median approach selected the median of MR estimates for causal estimation, while MR‐Egger regression allowed for estimating the intercept as a measure of average pleiotropy. MR‐PRESSO allowed for identifying the potential pleiotropic IVs and re‐estimation after excluding these outliers.^37^ TSMR analysis was performed to assess the effects of air pollutants on ALS. To account for multiple testing, the *p*‐value below the Bonferroni‐corrected threshold of 1.67E‐2 (0.05/3) was deemed as statistically significant.^80^

Sensitivity analyses were conducted to evaluate the robustness of the findings, including tests for heterogeneity and horizontal pleiotropy. Heterogeneity was assessed using Cochran's *Q* test, while horizontal pleiotropy was examined through MR‐PRESSO and MR‐Egger intercept test.^34^, ^81^ Although based on different assumptions, these tests fundamentally measured the extent to which the impact of one or more instrumental SNPs was exaggerated, not only through the hypothesized pathway but also through other unaccounted‐for causal pathways.

All statistical analyses were performed using R software. The “TwoSampleMR” package in R was utilized for data extraction, SNP clumping, harmonization, and TSMR.^82^

#### 2.3.2 Transcriptome‐wide association (TWAS) analysis and joint/conditional tests

To conduct transcriptomic imputation, we employed the FUSION method,^38^ which involved converting GWAS data into TWAS format. In this approach, a linear model based on expression quantitative trait loci was utilized to predict gene expression levels using the RNA‐seq of Genotype‐Tissue Expression version 8 (GTEx v8) (*N* = 183),^39^ CommonMind Consortium's (*N* = 452), and splicing (*N* = 452) reference^40^ as the reference panels of brain. Genes that exhibited significant associations with ALS were first selected. Then, among these ALS‐associated genes, the genes showed significant associations with air pollutants and were identified as potential mechanisms of air pollution to ALS. Bonferroni correction was conducted to account for multiple TWAS tests. The *p*‐value in TWAS below 0.05 but higher than the Bonferroni‐corrected *p*‐value was deemed to be a suggestive association.

To test how much GWAS signal and TWAS genes remain in a locus after the association of the significant genes in TWAS is removed, we performed joint/conditional tests in FUSION by FUSION.post_process and FUSION.assoc_test.

#### 2.3.3 Protein interaction and network prediction

We used GeneMANIA (http://genemania.org/) to predict the protein–protein interaction (PPI) of the genes that are significant in TWAS analysis.^41^ Detailed information on the included data sets in GeneMANIA is described somewhere else.^41^

## 3 RESULTS

After a strict filter, 14, 20, and 19 SNPs were selected as the IVs for PM2.5, NO_2_, and NO_X_ (Tables S2–S4). The *F*‐statistics were all above 10, showing strong robustness for the representation of the exposures.

**FIGURE 2:**
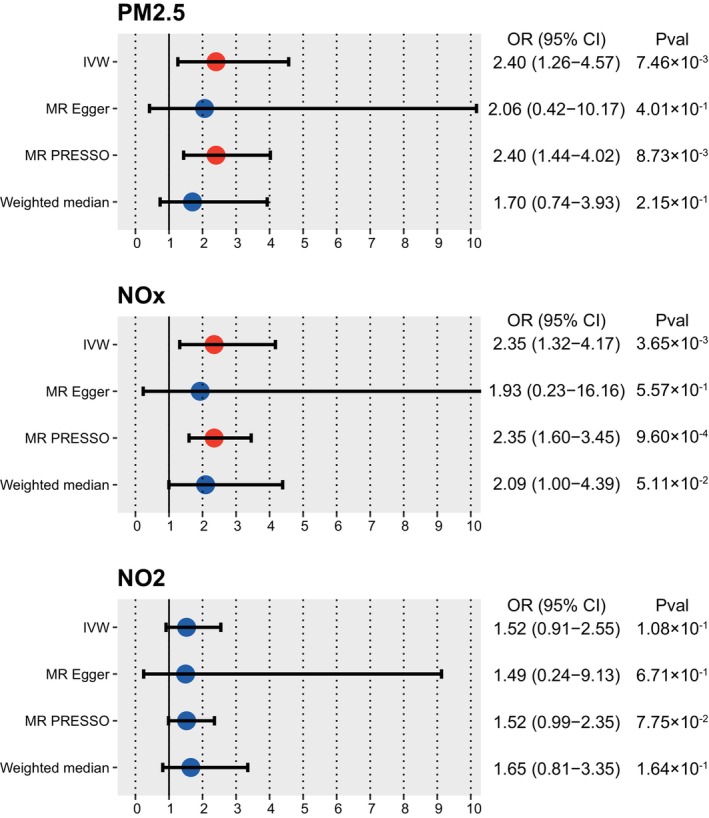
Forest plots illustrating the two‐sample Mendelian randomization (TSMR) estimates of the effects of air pollutants (PM2.5, NO_X_, and NO_2_) on the risk of amyotrophic lateral sclerosis (ALS). Each circle represents an individual instrumental variant (IV) with the corresponding odds ratio (OR) and 95% confidence interval (CI) indicated by the horizontal line. The *p*‐value indicates the statistical significance of the association between exposure and ALS risk. IVW (inverse variance weighting) was used as the primary method, with additional methods (weighted median, MR‐Egger, and Mendelian Randomization Pleiotropy RESidual Sum and Outlier [MR‐PRESSO]) employed as supplementary analyses.

We found that exposure to the higher level of PM2.5 genetically increased the risk of ALS (IVW, OR: 2.40 (95% CI: 1.26–4.57), *p* = 7.46E‐3) (Figure 2, Figure S1). MR PRESSO confirmed this effect of PM2.5 on ALS (OR: 2.40 [95% CI: 1.44–4.02], *p* = 8.73E‐3). This trend was also demonstrated in MR Egger and weighted median, although without significance (*p* > 0.05). Besides, higher exposure to NO_X_ was also found to genetically associate with a higher risk of ALS (IVW, OR: 2.35 [95% CI: 1.32–4.17], *p* = 3.65E‐3) (Figure 2 and Figure S2), with validation of MR PRESSO method (OR: 2.35 [95% CI: 1.60–3.45], *p* = 9.60E‐4). However, the effects of NO_2_ tended to be insignificant (*p* > 0.05) (Figure 2 and Figure S3).

Altogether, our analyses suggested a causal link between higher exposure to PM2.5, NO_X_, and increased risk of ALS, whereas NO_2_ had no causal effects on ALS.

For the sensitivity analyses, both MR PRESSO and MR egger showed no significant pleiotropy in all TSMR analyses (*p* > 0.05) (Table 1). Both MR egger and IVW in Cochran's Q test also showed no significant heterogeneity in all TSMR (*p* > 0.05) (Table 1). Therefore, our selected IVs and TSMR results showed great robustness.

**TABLE 1:** Sensitivity analyses for two‐sample Mendelian randomization.

Exposure	Heterogeneity		Pleiotropy	
	Method	*p* Value	Method	*p* Value
PM2.5	MR Egger	0.68	MR Egger	0.84
PM2.5	IVW	0.76	MR PRESSO	0.78
NO_X_	MR Egger	0.91	MR Egger	0.85
NO_X_	IVW	0.94	MR PRESSO	0.95
NO_2_	MR Egger	0.70	MR Egger	0.98
NO_2_	IVW	0.77	MR PRESSO	0.78

**FIGURE 3:**
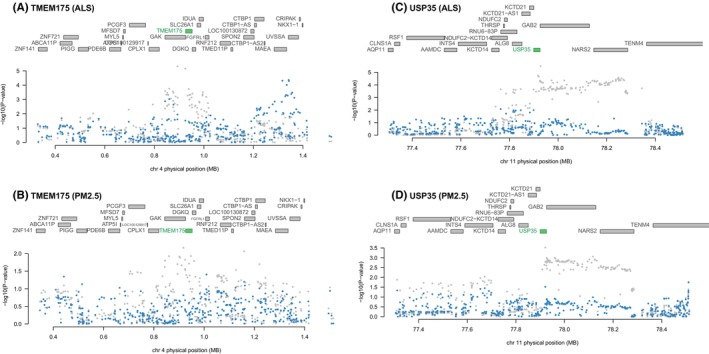
Joint/conditional plots of TWAS. All the genes in the locus were shown in the top panel. The genes that show a marginal association with TWAS are marked in blue, while the genes that exhibit a joint significance are highlighted in green. The lower panel displays a Manhattan plot illustrating the GWAS data before (gray) and after (blue) conditioning on the green genes. The GWAS signals dropped after conditioning the predicted expression of TMEM175(A,B) and USP35(C,D). GWAS, genome‐wide association study; TWAS, transcriptome‐wide association.

**FIGURE 4:**
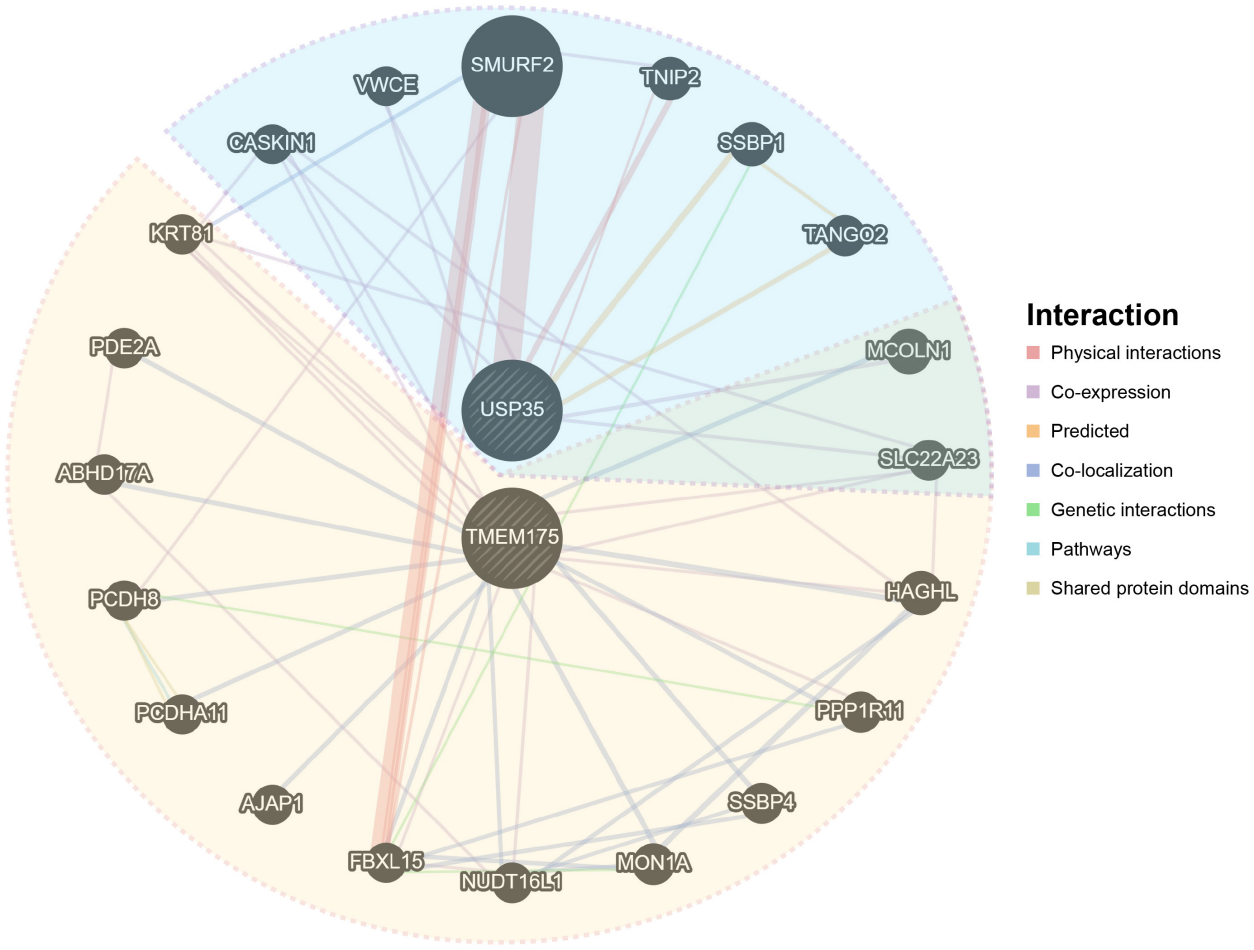
Protein–protein interaction plot for USP35 and TMEM175.

To investigate the potential mechanism of air pollutants‐inducing ALS, we conducted a TWAS analysis. In total, eight genes were significantly associated with ALS in TWAS and exhibited the same direction with PM2.5/NO_X_ among the panels of 9130 genes (*p* < 5.48E‐6, 0.05/9130) (Table 2 and Table S5). For PM2.5, USP35 and TMEM175 were significantly associated with the phenotype of higher exposure to PM2.5 (*p* < 6.23E‐3, 0.05/8). The joint/conditional tests showed that USP35 and TMEM175 were independently and strongly associated with ALS and PM2.5 in the corresponding locus (Figure 3). After excluding these two genes, the GWAS signal dropped. These results suggested that air pollutants might induce ALS through pathways related to USP35 and TMEM175. Then, we performed PPI analysis to identify the protein potentially interacting with USP35 and TMEM175 (Figure 4). USP35 was predicted to interact with CASKIN1, VWCE, SMURF2, TNIP2, SSBP1 and TANGO2, suggesting its involvement in diverse cellular functions. In contrast,KRT81, PDE2A, ABHD17A, PCDH8, PCDHA11, AJAP1, FBXL15, NUDT16L1, MON1A, SSBP4, PPP1R11 and HAGHL were predicted to interact with TMEM175. Specifically, MCOLN1 and SLC22A23 have been predicted to interact with both USP35 and TMEM175, highlighting their potential role in mediating interactions between these proteins.

**TABLE 2:** TWAS results for shared genes between ALS and air pollutants.

ID	Chr	Start	End	ALS		PM2.5		NO_X_	
				TWAS.Z	TWAS.P	TWAS.Z	TWAS.P	TWAS.Z	TWAS.P
USP35	11	77,899,857	77,925,757	4.66	3.17E−06	2.91	3.59E−03	2.35	1.90E−02
TMEM175	4	926,261	952,443	−4.73	2.24E−06	−2.82	4.79E−03	−2.53	1.15E−02
C9orf72	9	27,546,542	27,573,864	−11.92	9.41E−33	−2.27	2.31E−02	−2.23	2.61E−02
RQCD1	2	219,433,302	219,461,158	4.63	3.59E−06	1.73	8.44E−02	1.18	2.38E−01
GPX3	5	150,399,998	150,408,554	−6.00	1.97E−09	−1.11	2.69E−01	−1.32	1.88E−01
GGNBP2	17	36,544,887	36,544,888	4.81	1.50E−06	0.62	5.35E−01	0.73	4.67E−01
MYO19	17	34,851,598	34,891,305	−5.13	2.94E−07	−0.26	7.94E−01	−0.95	3.43E−01
RANBP10	16	67,806,651	67,806,652	4.56	5.11E−06	0.22	8.26E−01	0.66	5.11E−01

Abbreviations: ALS, amyotrophic lateral sclerosis; TWAS, transcriptome‐wide association.

Meanwhile, for NO_X_, USP35 and TMEM175 only showed suggestive association in the TWAS analysis. Interestingly, C9orf72, as the identified risky gene for ALS, showed significant association with ALS (*p* = 9.41E‐33) and suggestive association with both PM2.5 (*p* = 2.31E‐2) and NO_X_ (*p* = 2.61E‐2), indicating that C9orf72 might also exert certain effects from air pollutants to ALS. ALS5 (SPG11), another causative gene for ALS, showed trends of association with exposure to PM2.5 (*p* = 0.14) and NO_X_ (*p* = 0.26) (Table S5).

## 4 DISCUSSION

We used TSMR to investigate potential causal links between air pollution (including PM2.5, NO_X_, and NO_2_) and ALS. We found that higher exposure to PM2.5 and NO_X_ might be causally associated with increasing the risk of ALS. The relationship between NO_2_ and ALS showed a little positive trend but did not prove statistically significant. We also revealed that USP35 and TMEM175 potentially played important roles in air pollutants‐inducing ALS.

PM2.5 is an airborne particle in the atmosphere with a diameter of 2.5 μm or less that can be breathed by people.^42^ PM2.5 was conventionally considered to lead to many respiratory diseases after inhaling.^43^, ^44^ Recently, mounting evidence increasingly suggests that exposure to PM2.5 also impaired the central nervous system.^45^, ^46^ With a diameter of <2.5 μm, this fine particulate matter can reach the brain from the nasal cavity via two main pathways: directly penetrating olfactory epithelium or entering the circulation after traveling deep into the lungs and traversing the blood–brain barrier (BBB).^47^, ^48^ First, PM2.5 can cause mitochondrial dysfunction, traditionally considered one of ALS's four major pathophysiological mechanisms, including elevated production of reactive oxygen species (ROS) and reduced mitochondrial membrane potential.^49^, ^50^ The former affects the electron transport chain's electron transfer, whereas the latter promotes oxidative stress, resulting in neuron cell death and BBB dysfunction.^48^ Several studies suggested that PM2.5 exposure may aggravate ALS and other neurodegenerative diseases by causing neuroinflammation, oxidative stress, mitochondrial dysfunction, and neuronal damage.^51^, ^52^, ^53^ A meta‐analysis including 26 studies conducted reported a significant association between long‐term PM2.5 exposure and stroke, dementia, Alzheimer's disease, ASD, and Parkinson's disease.^54^ For the first time, we have identified significant causality between exposure to PM2.5 and the occurrence of ALS, built upon population‐based genetic analyses.

NO_X_ is a group of gases primarily emitted from combustion processes, such as emissions from vehicles and industry, which are widely reported to have detrimental effects on human health.^55^ Currently, several observational studies reported that long‐term exposure to NO_X_ was associated with a higher risk of ALS. However, there is still a lack of causality inference and experimental investigation, although NO_X_ exposure is associated with numerous ALS‐related pathways,^56^ such as oxidative stress^57^ and neuronal death.^58^, ^59^ In this study, we reported for the first time that NO_X_ might be causally associated with ALS risks.

Our investigation of the transcriptomic relationship between air pollutants and ALS revealed that TMEM175 and USP35 might intermediate from PM2.5 to ALS. TMEM175 is a lysosomal ion channel that assists in the digestion of abnormal proteins and mitochondrial homeostasis. Dysfunction of TMEM175 was correlated with multiple neurologic disorders.^60^ For example, deficiency in TMEM175 could cause neuron death, motor impairment, and Parkinson's disease.^61^ In the brain of ALS, the level of TMEM175 was also reported to be abnormally decreased, which was consistent with our results.^62^ Meanwhile, the downstream of TEME175, including homeostasis of protein and mitochondria, is the typical pathophysiology of ALS.^56^ However, the biological effects of TMEM175 exerted in ALS and how PM2.5 influences the function of TMEM175 need to be further explored in the laboratory.

USP35 is an enzyme of the deubiquitinase family, which removes ubiquitin molecules and regulates protein homeostasis. In ALS, deubiquitinase plays a pivotal role in the pathogenesis. The deubiquitinase could regulate proteotoxicity and control the protein quality, thus influencing the development of ALS. Inhibiting deubiquitinase could protect against proteotoxicity from ALS.^63^ Besides, USP35 could regulate PARK2‐mediated mitophagy and mitochondria quality control.^64^ Defects in mitochondria function are related to multiple ALS pathologic activities, including neuronal calcium homeostasis, autophagy, and axonal degeneration.^56^ Additionally, the proteins we predicted that interacted with USP35 have also been reported in ALS. For example, SMURF2 was reported to be immunopositive in ALS and co‐localize with TDP‐43, a known causative protein of ALS.^65^

C9orf72, widely regarded as the most common genetic cause of ALS,^66^ was significant in our ALS TWAS analysis. The mechanisms of C9orf72 inducing ALS were well elucidated in previous studies.^56^, ^66^ Our TWAS analysis found that the C9orf72 expression is suggestively associated with PM2.5 and NO_X_. Unfortunately, it did not pass Bonferroni's correction. Considering the importance of C9orf72 in ALS, this suggestive evidence should not be neglected. It is reported that PM2.5 may have an unclear mechanism for DNA methylation,^67^, ^68^ which acts as a gene silencer to suppress the production of certain DNA pieces, such as the C9orf72 expansion.^69^, ^70^ Thus, one potential epigenetic explanation is that PM2.5‐related demethylation of C9orf72 expansion induces the expression of RNA foci and DPR expression. Further exploration is needed to elucidate whether air pollutants could influence the level or function of C9orf72 and the underlying mechanisms involved.

ALS5 (SPG11) is the major gene causing autosomal recessive ALS.^71^ We found trends in the association of ALS5 with PM2.5 and NO_X_. Considering air pollutants might affect levels of multiple proteins,^72^ induce genetic mutations,^73^ and potentially cause ALS, studies with large sample sizes in the future might shift this trend into significance.

Limitations of our study should be taken into consideration. First, our findings need more experimental validation. Second, the data sets in this study consisted of European populations, limiting the generalizability to other ethnics. Last, as a context‐dependent and environment‐related GWAS, the IVs of air pollutants might not be the perfect proxy for intrinsic measurement. The LD‐score ratio of 53%–63% in air pollutants GWAS (collected from the IEU database) can be interpreted as certain proportion of signals in these GWAS coming from potential confounders, likely from population structure, rather than polygenic signals (Table S6). Three reasons underlay this potential limitation: (1) while the IVs of air pollution were statistically significant in the UK Biobank data set, their biological significance and transferablity needs to be further validated; (2) the measurement of air pollutant exposure in the GWAS is based on participant home address and may contain bias from home address change across lifetime; (3) the air pollutants GWAS are likely confounded by imperfectly corrected latent population structure which has been previously shown to affect GWAS in the UK Biobank in spite of stringent corrections^74^ and is strongly correlated with participant location. These issues could probably restrict the relevance and independence assumptions of MR analysis to a certain degree.^75^

To summarize, our study has established a causal relationship between exposure to air pollutants and ALS using MR analysis based on the largest and latest GWAS. Our findings suggest that PM2.5 and NO_X_ exposure is associated with an increased incidence of ALS, while NO_2_ exposure did not have a statistically significant effect. Through transcriptome‐wide association studies, we identified that TMEM175 and USP35 might intermediate from PM2.5 to ALS, related to the homeostasis of proteome and mitochondria.

Our study contributes to a growing body of evidence that environmental exposures, such as air pollution, may play a role in the development of ALS. These findings highlight the need for further research to validate our results and explore potential preventive measures for this devastating disease.

## AUTHOR CONTRIBUTIONS

Quan Cheng and Hao Zhang conceived and designed the research. Zhihao Li and Jie Wen wrote the first draft of the manuscript. Zhihao Li, Jie Wen, Wantao Wu, Ziyu Dai, Xisong Liang, and Nan Zhang contributed to data acquisition, data analysis, and interpretation. Zhihao Li, Jie Wen, Wantao Wu, Ziyu Dai, Xisong Liang, Nan Zhang, Quan Cheng, and Hao Zhang contributed to the revision of the paper. Quan Cheng and Hao Zhang provided funding support. All authors contributed to the article and approved the final manuscript.

## FUNDING INFORMATION

This study was funded by Chongqing Postdoctoral Research Special Funding Project (2023CQBSHTB3095), Chongqing Postdoctoral Science Foundation (CSTB2023NSCQBHX0002), China Postdoctoral Science Foundation (2023MD734131), and Hunan Youth Science and Technology Talent Project (2023RC3074).

## CONFLICT OF INTEREST STATEMENT

The authors declare no conflicts of interest.

## Supporting information


Data S1

## Data Availability

The data sets supporting the conclusions of this article are available in the UK Biobank from MRC IEU (https://gwas.mrcieu.ac.uk/), van Rheenen et al. GWAS meta‐analysis. Further data inquiries can be directed to the corresponding authors.
