# Supplementary figures and images for: Causal relationship and shared genes between air pollutants and amyotrophic lateral sclerosis: A large‐scale genetic analysis
Source: CNS Neurosci Ther. 2024 Jul 5;30(7):e14812. doi: 10.1111/cns.14812 (PMC11226412; doi:10.1111/cns.14812)

# MR Test

- Inverse variance weighted
- MR Egger
- Weighted median

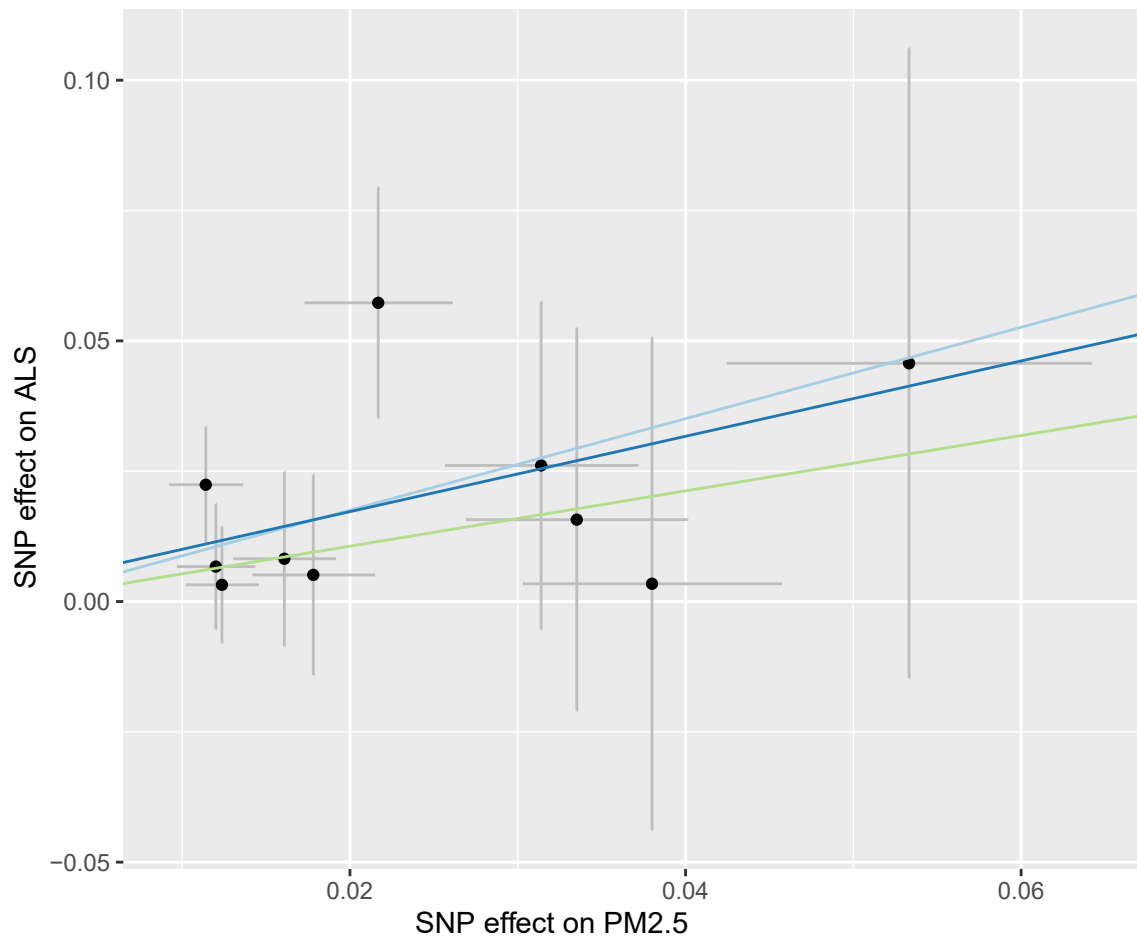

Supplement: Supplementary file 1 — Data S1 [file CNS-30-e14812-s001.zip › sFigure 1.pdf]

# MR Test

- Inverse variance weighted
- Weighted median
- MR Egger

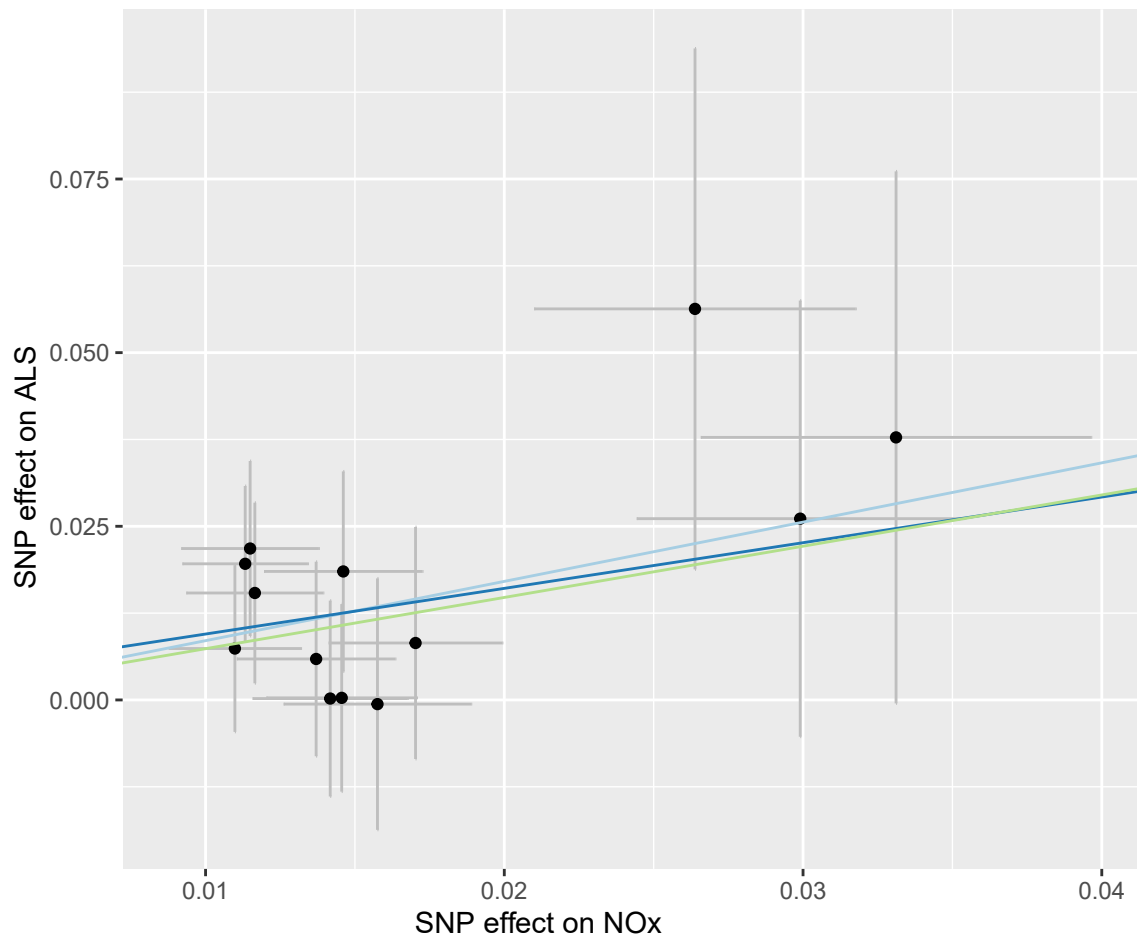

Supplement: Supplementary file 1 — Data S1 [file CNS-30-e14812-s001.zip › sFigure 2.pdf]

# MR Test

- Inverse variance weighted
- MR Egger
- Weighted median

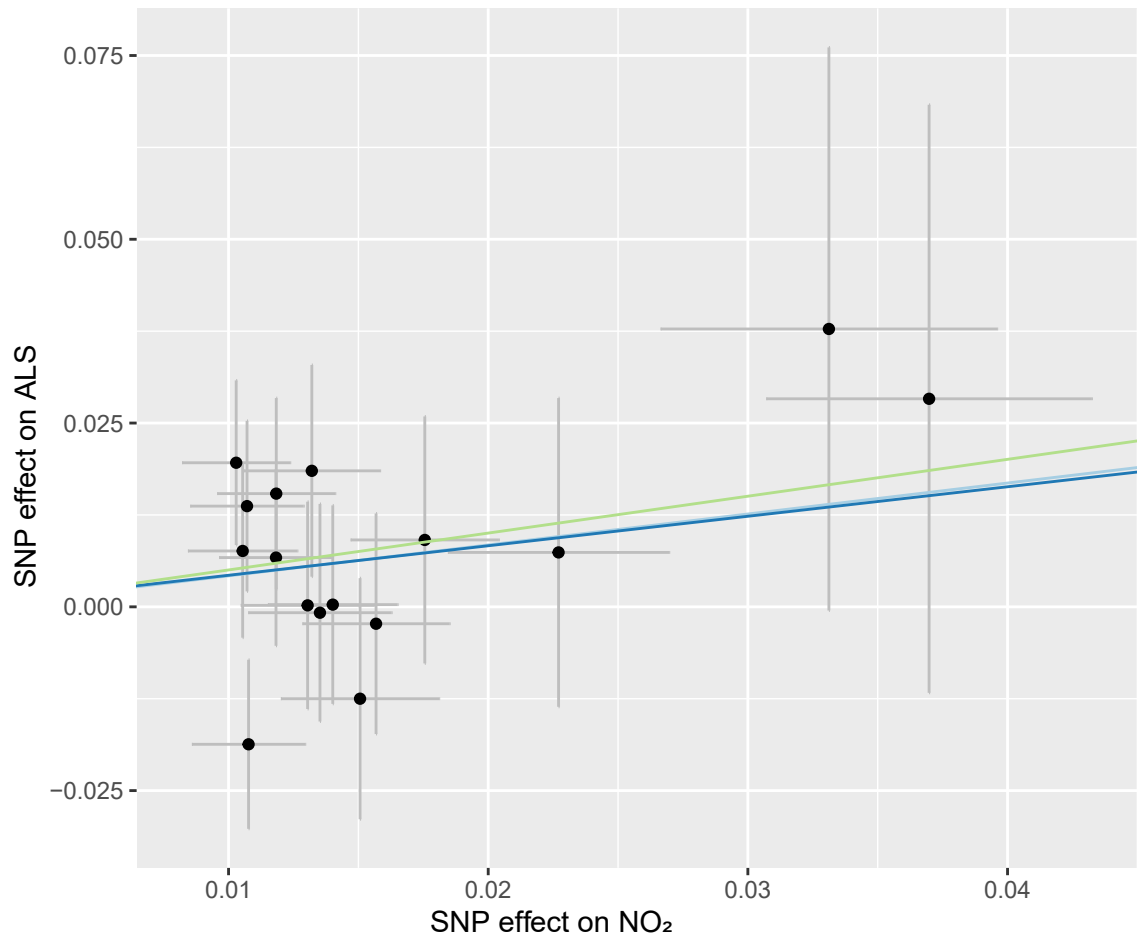

Supplement: Supplementary file 1 — Data S1 [file CNS-30-e14812-s001.zip › sFigure 3.pdf]
